# Supplementary material for: Multiscale Simulations Elucidate the Mechanism of Polyglutamine Aggregation and the Role of Flanking Domains in Fibril Polymorphism
Source: J Phys Chem B. 2025 Oct 15;129(43):11205–19. doi: 10.1021/acs.jpcb.5c06627 (PMC12581130; doi:10.1021/acs.jpcb.5c06627)
Supplement: Supplementary file 1 [file jp5c06627_si_001.pdf]

Supporting Information

Multiscale Simulations Elucidate the Mechanism of Polyglutamine Aggregation and the Role of Flanking Domains in Fibril Polymorphism

Avijeet Kulshrestha[1*], Tien Minh Phan[1*], Azamat Rizuan[1*], Priyesh Mohanty[1*] and Jeetain Mittal[1,2,3*]

[1]Artie McFerrin Department of Chemical Engineering, Texas A&M University, College Station, TX, 77843

[2]Department of Chemistry, Texas A&M University, College Station, TX, 77843

[3]Interdisciplinary Graduate Program in Genetics and Genomics, Texas A&M University, College Station, TX, 77843

1. Supporting Table.

Table S1: The comprehensive details of the simulations performed in our study.

System information	Initial (final state)	Number of chains	Simulation time (ns)
Q16 monomer	IDP (partial helix)	1	1000
All-atom Q16 (semi-infinite)	BT-A1 fibril (fibril)	16	1000
	BT-A2 fibril (fibril)	16	1000
	BT-A3 fibril (fibril)	16	1000
	BT-A4 fibril (fibril)	16	1000
	BA-A1 fibril (fibril)	16	1000
	BA-A2 fibril (fibril)	16	1000
	BA-A3 fibril (fibril)	16	1000
	BA-A4 fibril (fibril)	16	1000
Multi-eGO (10 mM Q16)	BT-A1 dilute (fibril)	1000	1000 × 3
	BA-A1 dilute (fibril)	1000	1000 × 3
Multi-eGO (1 mM Q16)	BT-A1 dilute (fibril)	1000	1000 × 3
	BA-A1 dilute (fibril)	1000	1000 × 3
Multi-eGO (0.5 mM Q16)	BT-A1 dilute (fibril)	1000	1000 × 3
	BA-A1 dilute (fibril)	1000	1000 × 3
Multi-eGO (0.25 mM Q16)	BT-A1 dilute (fibril)	1000	1000 × 3
	BA-A1 dilute (fibril)	1000	1000 × 3
Q16-HttEx1 monomer	IDP (partial helix)	1	1000
Q16-HttEx1 all-atom dense phase	Dense phase (partial helix)	170	5000
Multi-eGO (10 mM Q16-HttEx1)	BT-A1 dilute (fibril)	500	1000 × 3
	BA-A1 dilute (fibril)	500	1000 × 3
Multi-eGO (8 mM Q16-HttEx1)	BT-A1 dilute (fibril)	500	1000 × 3
	BA-A1 dilute (fibril)	500	1000 × 3

Multi-eGO (6 mM Q16-HttEx1)	BT-A1 dilute (fibril)	500	1000 × 3
	BA-A1 dilute (fibril)	500	1000 × 3
Multi-eGO (4 mM Q16-HttEx1)	BT-A1 dilute (fibril)	500	1000 × 3
	BA-A1 dilute (fibril)	500	1000 × 3
Multi-eGO (2 mM Q16-HttEx1)	BT-A1 dilute (fibril)	500	1000 × 3
	BA-A1 dilute (fibril)	500	1000 × 3
Multi-eGO (1 mM Q16-HttEx1)	BT-A1 dilute (fibril)	500	1000 × 3
	BA-A1 dilute (fibril)	500	1000 × 3
Multi-eGO (0.5 mM Q16-HttEx1)	BT-A1 dilute (fibril)	500	1000 × 3
	BA-A1 dilute (fibril)	500	1000 × 3
Multi-eGO (0.25 mM Q16-HttEx1)	BT-A1 dilute (fibril)	500	1000 × 3
	BA-A1 dilute (fibril)	500	1000 × 3
All-atom simulation of Q16-HttEx1 protofibril	Fibril (fibril)	19	1000
	Fibril (fibril)	22	1000

2. Supporting Figures

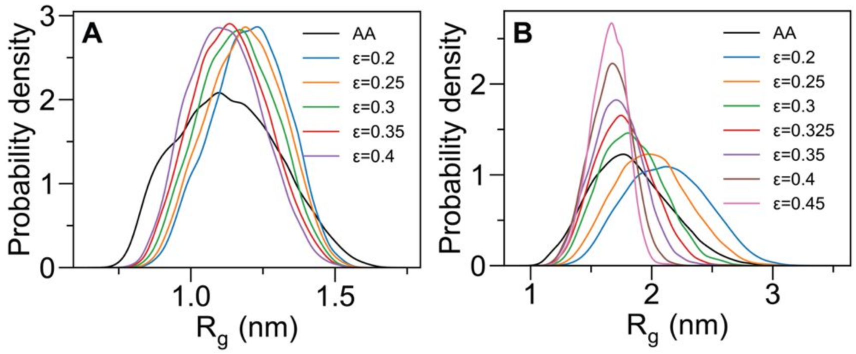

Figure S1: **Parameterization of Multi-eGO models against the AA monomeric ensemble**. Radius of gyration (R_g) distribution for varying values of the free parameter (ε) in Multi-eGO simulation of (**A**) the Q16 peptide and (**B**) the Q16-HttEx1 protein, compared to the corresponding AA simulations.

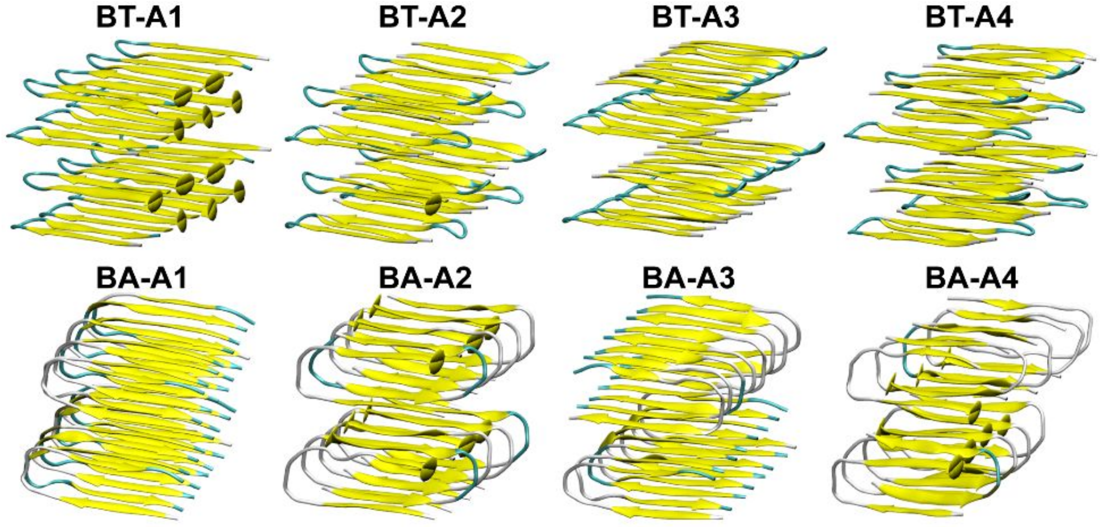

Figure S2: **Fibril arrangements considered in the study**. BT-A1: β-turn with the end-termini aligned in the same direction. BT-A2: β-turn with the end-termini arranged alternately along the lateral direction. BT-A3: β-turn with the end-termini arranged alternately along the axial direction. BT-A4: β-turn with the end-termini arranged alternately in both lateral and axial directions. Similarly, BA-A1, BA-A2, BA-A3, and BA-A4 represent the corresponding directional arrangements for the β-arc polyQ model.

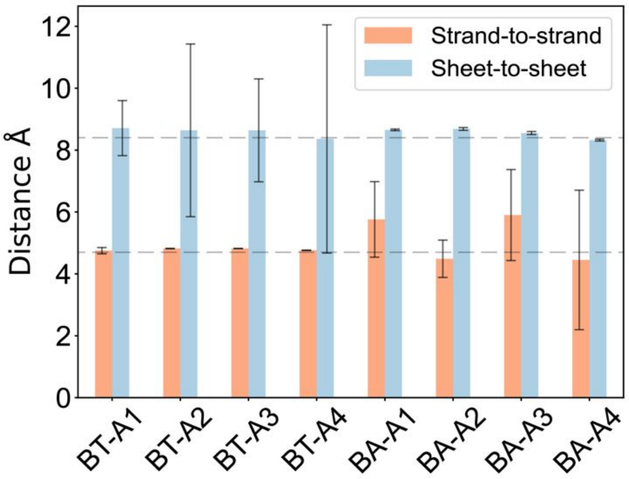

Figure S3: **Distance analysis of fibril arrangements**. Sheet-to-sheet and strand-to-strand distances for all directional arrangements considered in this study (Figure S2). The dashed lines represent distances measured from X-ray diffraction experiments: 4.75 Å for strand-to-strand spacing and 8.3 Å for sheet-to-sheet spacing.(6)

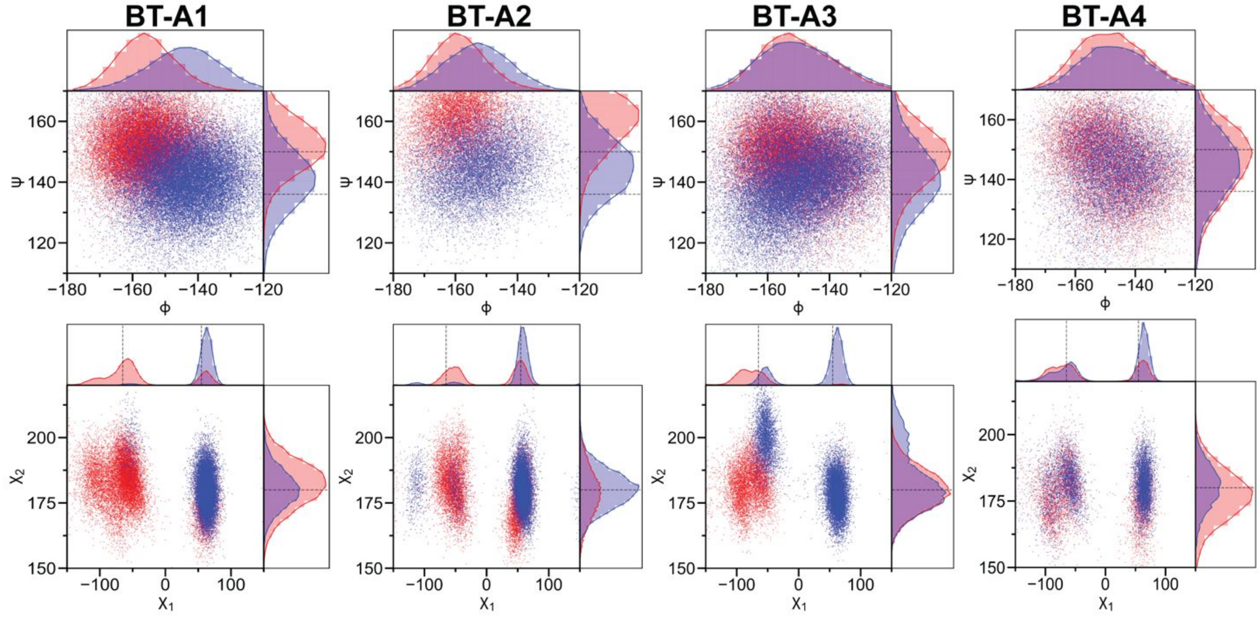

Figure S4: **Dihedral angle distributions of β-turn fibril arrangements**. Backbone (ϕ and ψ) and Side chain (χ1 and χ2) dihedral angle distributions for β-turn configurations shown in Figure S2. Black dashed lines indicate values observed in ssNMR experiments.(6)

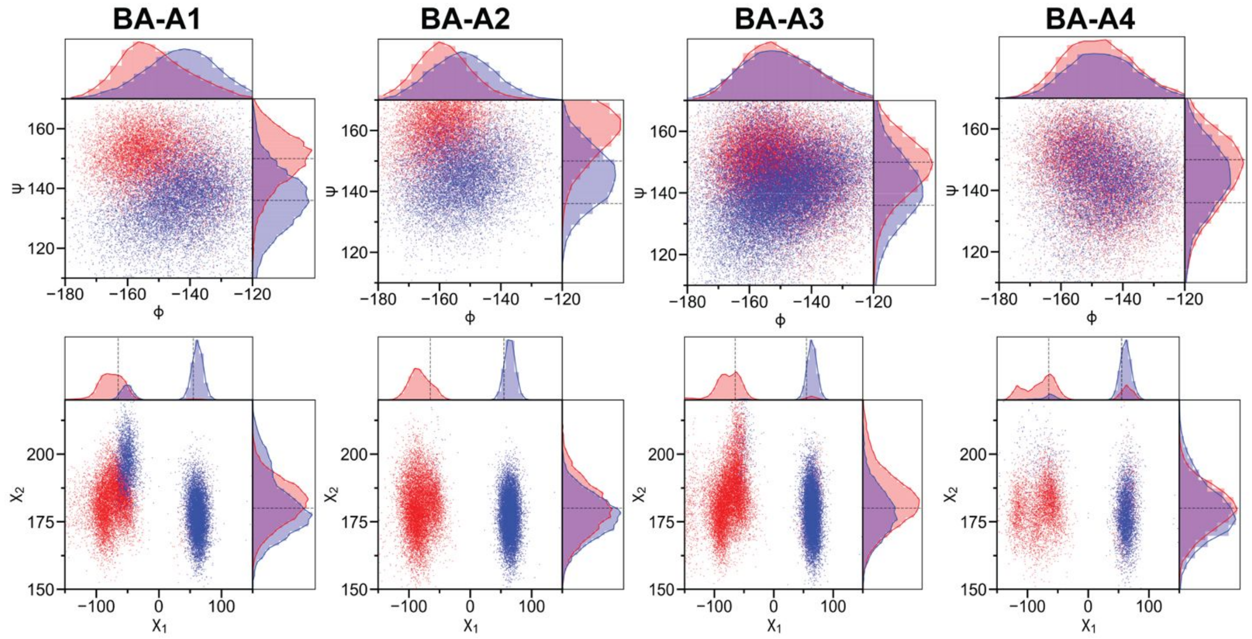

Figure S5: **Dihedral angle distributions of β-arc fibril arrangements**. Backbone (ϕ and ψ) and Side chain (χ1 and χ2) dihedral angle distributions for β-arc configurations shown in Figure S2. Black dashed lines indicate values observed in ssNMR experiments.(6)

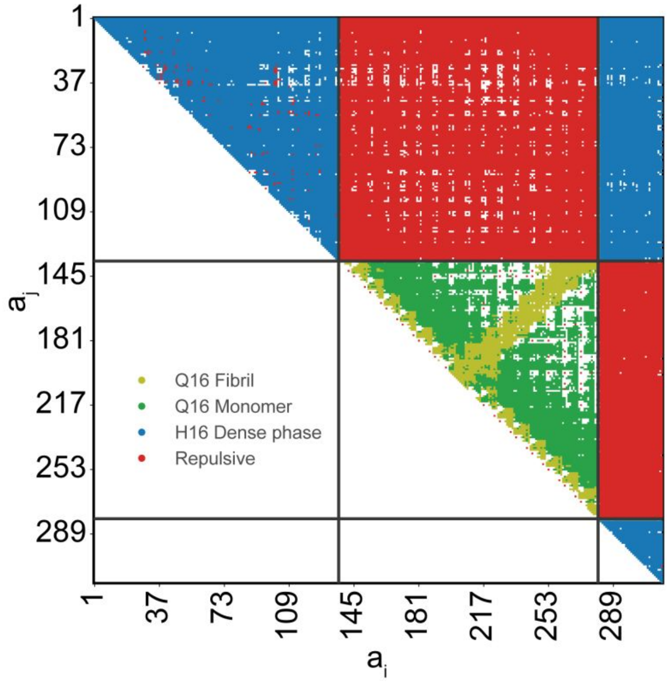

Figure S6: **Contact matrix for Q16-HttEx1 Multi-eGO Model.** All heavy atoms Multi-eGO Q16-HttEx1 model contact matrix incorporates contacts for the N17 and P5 flanking domains derived from the dense phase, while the contacts for Q16 are based on the Q16 Multi-eGO model. Interactions between the flanking domains and Q16 are designed to be purely repulsive to reduce frustration in the landscape.

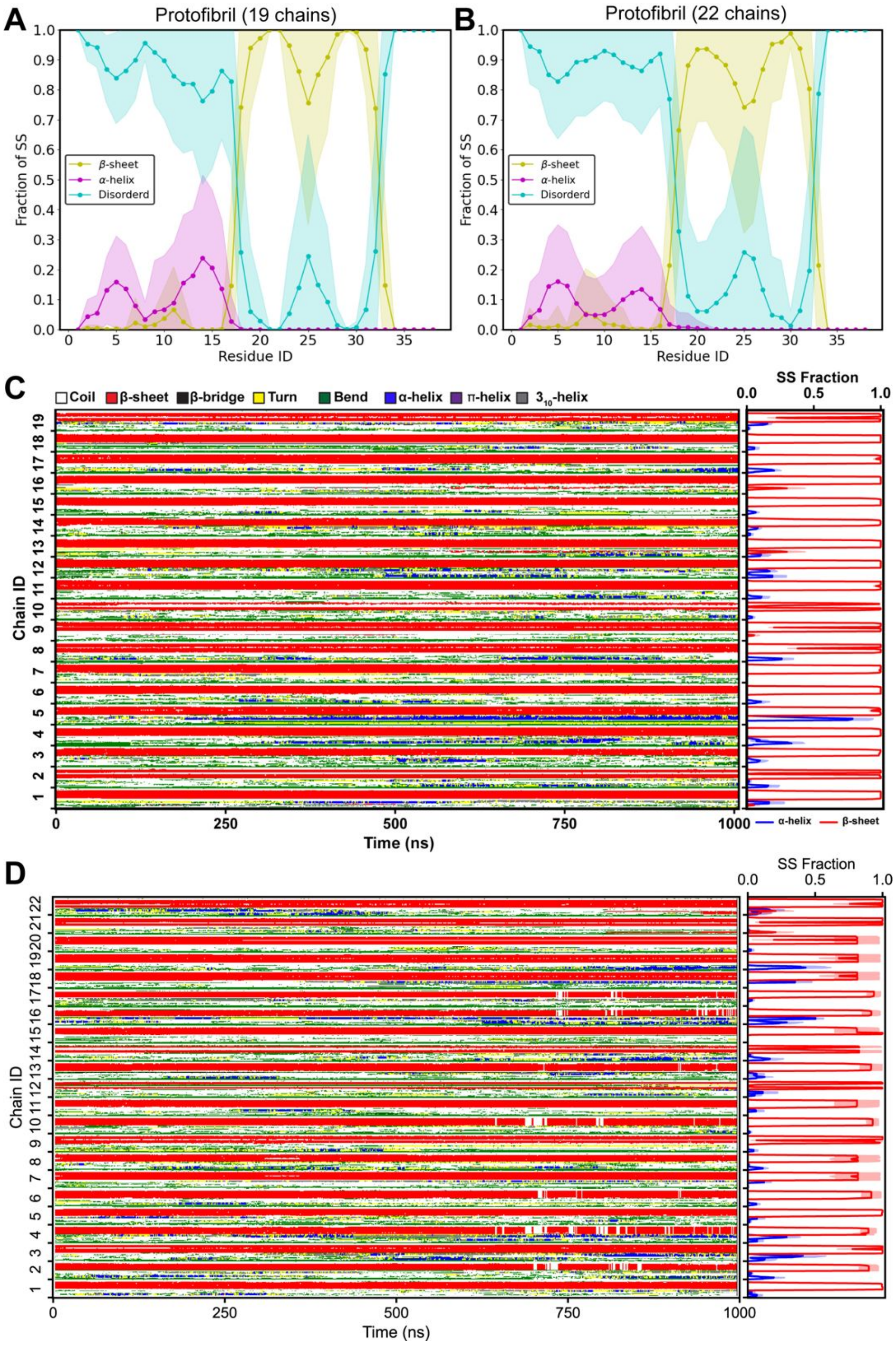

Figure S7: **Structural Characterization of Q16-HttEx1 Protofibrils from All-Atom Simulations.** (**A, B**) The average fraction of secondary structure content for tow selected protofibrils of different sizes (19 and 22 chains, respectively), with the shaded region representing the standard deviation. (**C, D**) Time-dependent secondary structure variation of all chains in the two protofibrils containing (**C**) 19 chains and (**D**) 22 chains, demonstrating stable β-sheet structures in the polyQ core and dynamic conformational changes in the N17 and P5 regions.

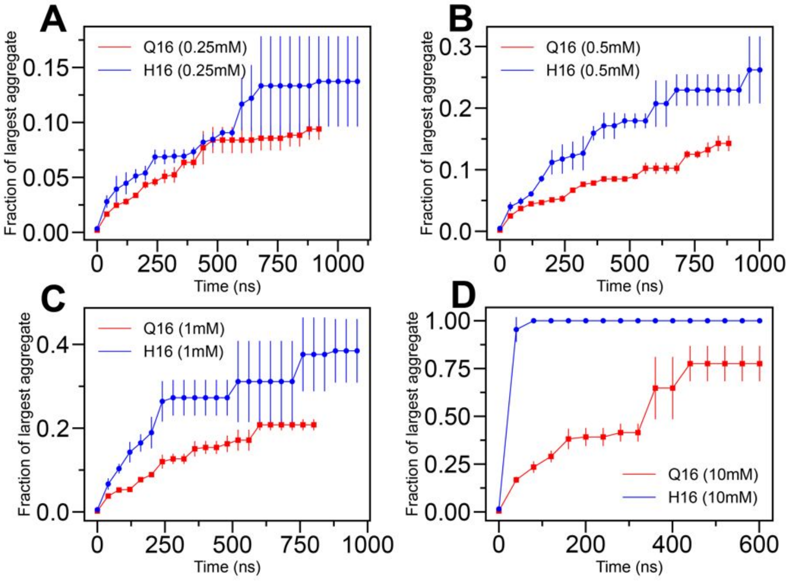

Figure S8: **Aggregation kinetics of Q16 and Q16-HttEx1.** Time evolution of the maximum cluster fraction of Q16 and Q16-HttEx1 at (**A**) 0.25 mM, (**B**) 0.5 mM, (**C**) 1 mM, and (**D**) 10 mM concentrations and 300 K temperature.

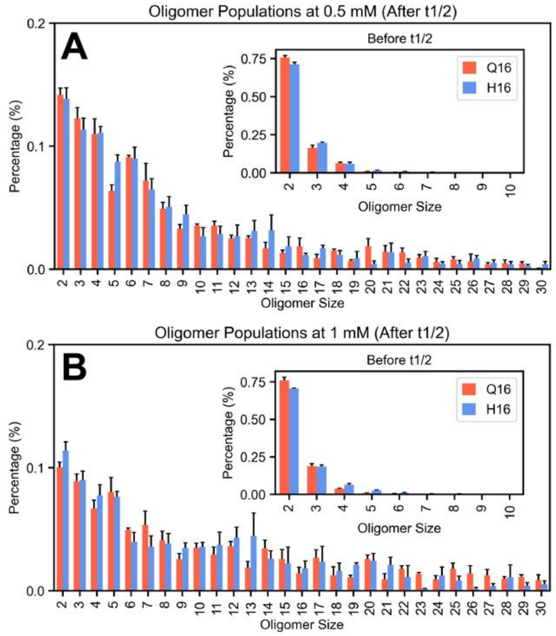

Figure S9: **Oligomer size distribution.** The oligomeric size distribution change before (inset) and after the half-life of monomer, the time at which the monomer fraction reduces by half, for (**A**) 0.5 mM and (B) 1 mM concentrations.

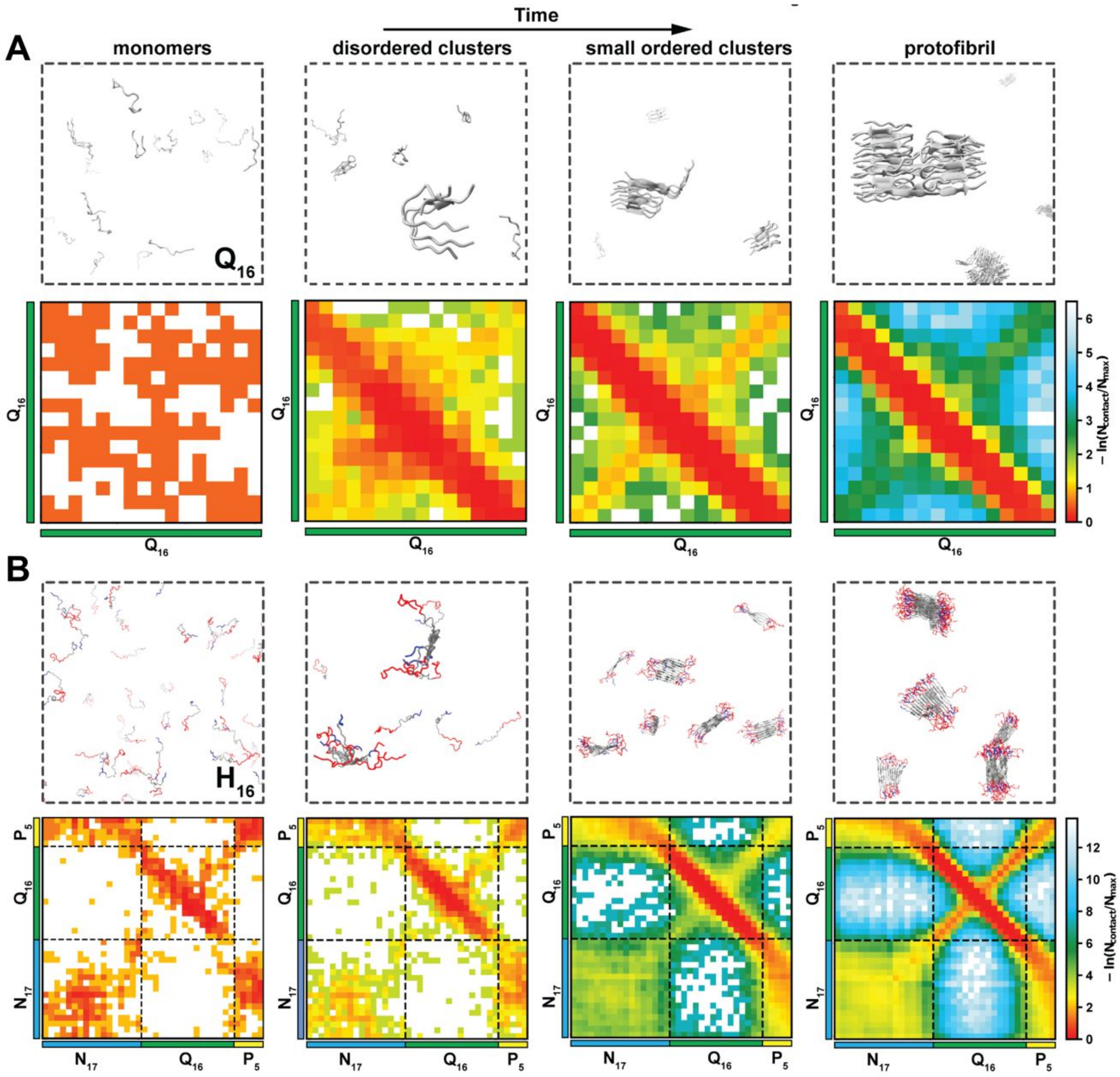

Figure S10: **Aggregation mechanism of Q16 and Q16-HttEx1.** Schematic representation the aggregation processes and evolution of intermolecular contacts for (**A**) Q16 and (**B**) Q16-HttEx1. Initially, monomers self-assemble into disordered clusters, which subsequently undergo structural rearrangements to form small, ordered clusters. These clusters serve as templates for further elongation, ultimately leading to protofibril formation.

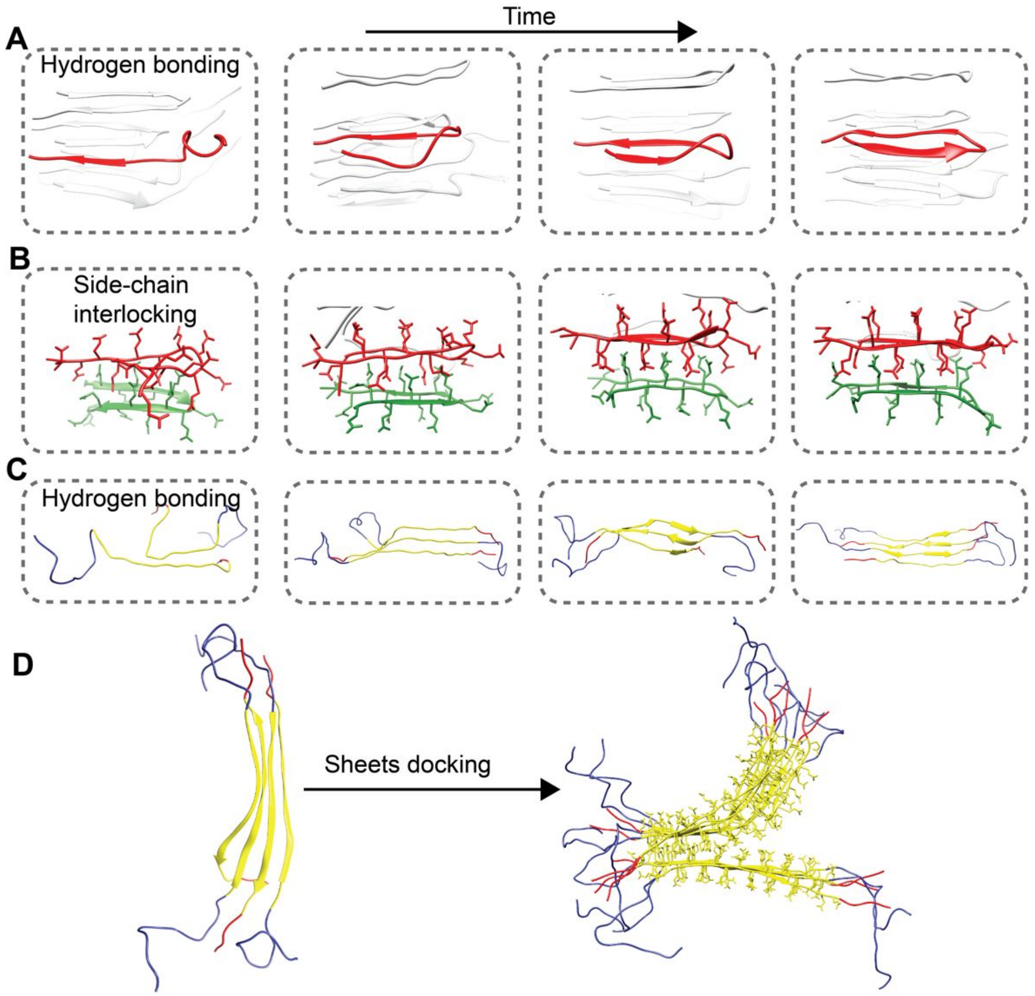

Figure S11: **Early stage aggregation mechanism of Q16 and Q16-HttEx1.** Two different types of dock-and-lock mechanisms observed in Q16 aggregation simulations – (**A**) Hydrogen bonding driven – the Q16 chain (red) docks through interchain hydrogen bonding, followed by full conversion to a β-sheet; and (**B**) Side-chain interlocking driven – chain-1 (green) initially exists in a β-turn conformation and serves as a template for chain-2 (red) to dock via its side chains. Once chain-2 fully docks, the side chains of both chains rearrange cooperatively, causing chain-1 to lose its β-turn structure. Ultimately, this cooperative motion allows both molecules to achieve proper side-chain interdigitation in a β-turn configuration. (**C**) Q16-HttEx1 aggregation is initiated by Q16 domain (yellow) interchain interaction, leading to the formation of β-sheets. (**D**) Subsequently, these β-sheets dock onto other β-sheets through side-chain interlocking to produce a protofibril.

3. Supporting Movies

Movie S1. All-atom molecular dynamics (AAMD) simulation of an H16 protofibril composed of 19 chains. The structure is color-coded by secondary structure: α-helix (purple), β-sheet (yellow), and coil/turn/bend (silver).

Movie S2. AAMD simulation of an H16 protofibril composed of 22 chains. The structure is color-coded by secondary structure: α-helix (purple), β-sheet (yellow), and coil/turn/bend (silver).

Movie S3. Multi-eGO simulation of a Q16 system containing 1000 chains. Chains are color-coded individually to distinguish different molecules.

Movie S4. Multi-eGO simulation of an H16 system containing 500 chains. Domains are color-coded as follows: N17 = red, Q16 = silver, and P5 = blue.
